# Pitch of Harmonic Complex Tones: Rate Coding of Envelope Repetition Rate in the Auditory Midbrain

**DOI:** 10.3813/AAA.919239

**Published:** 2018

**Authors:** Yaqing Su, Bertrand Delgutte

**Affiliations:** 1) Eaton-Peabody Labs, Massachusetts Eye & Ear, Boston, MA, USA.; 2) Dept. of Biomedical Engineering, Boston University, Boston, MA, USA; 3) Dept. of Otolaryngology, Harvard Medical School, Boston, MA, USA

## Abstract

Envelope repetition rate (ERR) is an important cue for the pitch of harmonic complex tones (HCT), especially when the tone consists entirely of unresolved harmonics. Neural synchronization to the stimulus envelope provides a prominent cue for ERR in the auditory periphery, but this temporal code becomes degraded and gives way to rate codes in higher centers. The inferior colliculus (IC) likely plays a key role in this temporal-to-rate code transformation. Here we recorded single IC neuron responses to HCT at varying fundamental frequencies (*F*_0_). ERR was manipulated by applying different inter-harmonic phase relationships. We identified a subset of neurons that showed a ‘non-tonotopic’ rate tuning to ERR between 160 and 1500 Hz. A comparison of neural responses to HCT and sinusoidally amplitude modulated (SAM) noise suggests that this tuning is dependent on the shape of stimulus envelope. A phenomenological model is able to reproduce the non-tonotopic tuning to ERR, and suggests it arises in the IC via synaptic inhibition.

## 1. Introduction

Harmonic complex tones present in speech, musical sounds and animal vocalizations evoke a strong pitch sensation at their fundamental frequency (*F*_0_), even if they contain no energy at *F*_0_ (“missing fundamental”). Although pitch plays important roles in speech and music perception and in the perceptual organization of auditory scenes, the neural mechanisms underlying pitch perception are still poorly understood. On the one hand, studies of the auditory nerve (AN) and cochlear nucleus (CN) have described multiple potential codes to pitch cues, including a rate-place code for resolved harmonics, temporal codes based on interspike interval distributions, and spatio-temporal codes dependent on both cochlear frequency selectivity and neural phase locking [1, 2, 3, 4]. On the other hand, pitch-selective neurons have been identified in a specific region of marmoset auditory cortex [5].

However, little is known about how the peripheral representations of pitch cues are integrated and transformed into a cortical pitch code. IC, the major nucleus in the auditory midbrain, is a logical target for addressing this question because it is the site of a transformation from a temporal code to a rate code for the frequency of amplitude modulations [6, 7].

Pitch percepts can arise either through the tonotopic pattern of low-numbered harmonics that are individually resolved by the cochlea or through neural phase locking to the envelope periodicity resulting from beating of unresolved harmonics encompassed by one auditory filter [8]. In general, the pitch produced by unresolved harmonics is less salient and more dependent on the inter-harmonic phase relationships than the pitch produced by resolved harmonics [8, 9].

Here, we investigate the neural representation of pitch cues by single-neuron recording from the IC in unanesthetized rabbits. Rabbits have good low frequency hearing like humans [10] and can discriminate *F*_0_ of HCTs with missing fundamentals [11]. We characterize a rate code for the ERR that is unrelated to pure-tone frequency tuning and is likely derived from temporal cues in the auditory periphery. This “non-tonotopic” rate code could play a role in pitch perception based on unresolved harmonics.

## 2. Methods

### 2.1. Single-unit Recording

Extracellular spiking activity of 145 single IC neurons was recorded from 4 head-fixed, unanesthetized rabbits. All procedures were approved by the Animal Care Committee of Massachusetts Eye and Ear. Details of the preparation can be found in [12].

### 2.2. Stimuli

Acoustic stimuli were generated in MATLAB and diotically delivered by earphones coupled through custom- fitted ear molds. All stimuli were presented 10 times in random order, 200-ms on, 300-ms off.

**Figure 1.**
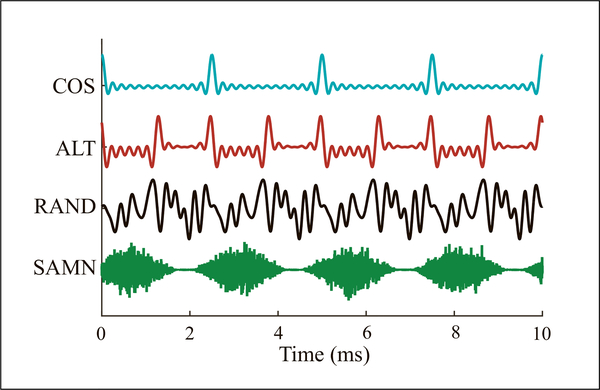
Temporal waveforms of HCT with three inter-phase relationships at *F*_0_ = 400 Hz, and SAM noise at *F*_*m*_ = 400 Hz. COS: ERR = *F*_0_, ALT: ERR = 2*F*_0_, RAND: flat envelope, SAMN: ERR = *F*_*m*_, and envelope shape different from HCT.

To manipulate the shape and repetition rate of the temporal envelope, HCT consisting of equal-amplitude harmonics up to 18 kHz and with F_0_ ranging from 26 to 2560 Hz were generated with three phase relationships among harmonics (Figure 1): 1) COS: all harmonics in cosine phase, ERR = *F*_0_; 2) ALT: even harmonics in cosine phase and odd harmonics in sine phase, ERR = 2*F*_0_; 3) RAND: harmonics in random phase to minimize envelope fluctuations. The overall sound level was 40 dB SPL for all HCTs.

In 66 of the 145 neurons, SAM broadband noise (SAMN) was interleaved with HCTs at 40 dB SPL. Modulation frequencies (*F*_m_) matched the *F*_0_s of the HCTs. The modulation depth was always 1.

### 2.3. Data Analysis

**Figure 2.**
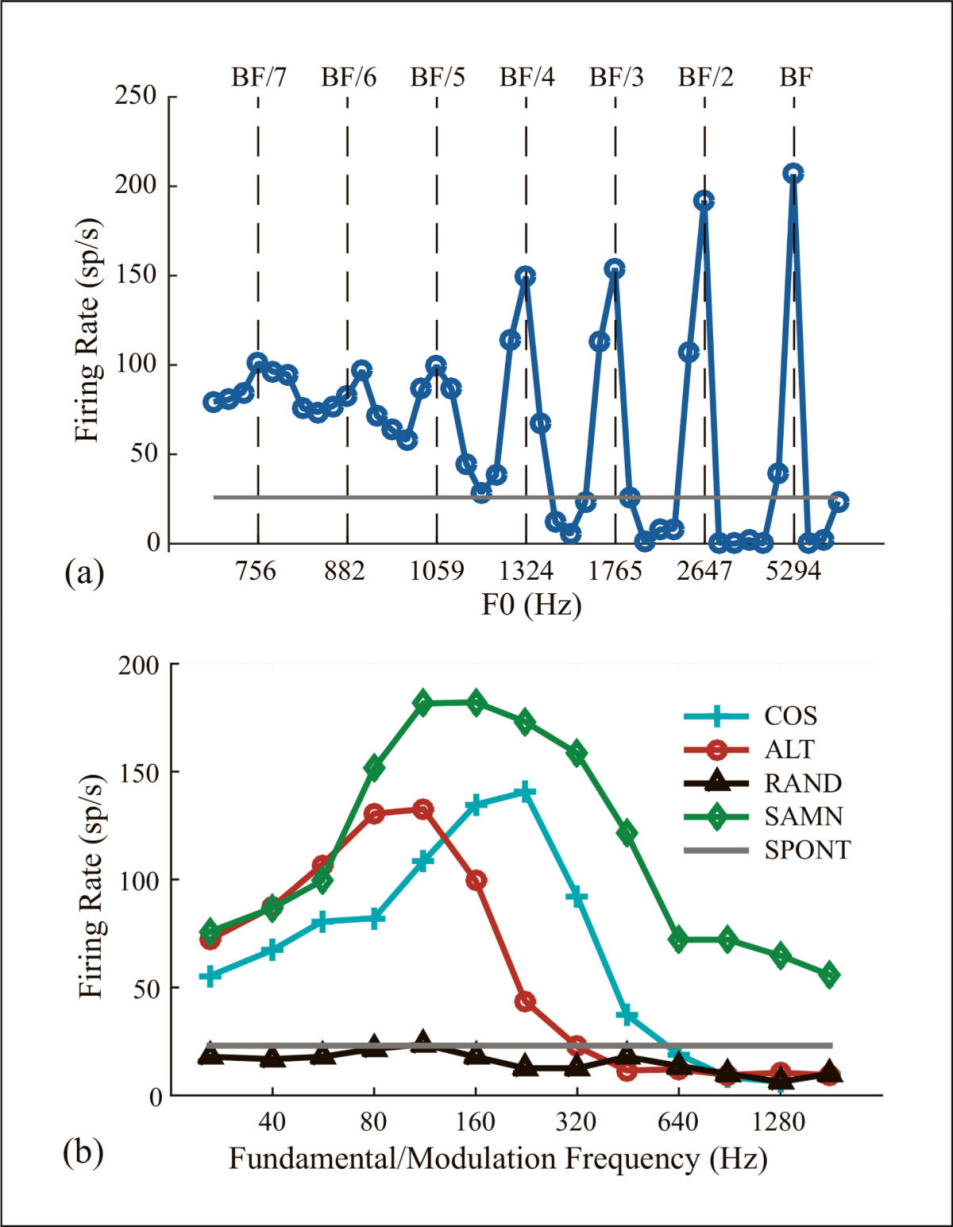
Firing rate profiles of two neurons tuned to different attributes of the stimuli. (a) Rate profile of the neuron showed local maxima at BF/n, n = 1, 2, …, 5. (b) Neuron (BF = 500 Hz) showed BP tuning to ERR of COS and ALT HCT, and SAMN. Best ERR is 190 Hz for COS, 129 Hz for SAMN.

For every neuron, we measured the average firing rate over the stimulus duration at each *F*_0_ or *F*_*m*_ to create a rate- frequency profile (Figure 2). Spontaneous rate was averaged over the last 200 ms of the inter-stimulus interval. The neuron’s best frequency (BF) was defined as the pure tone frequency that elicited the maximum firing rate at ~40dB SPL.

### 2.4. SFIE Model

We implemented the Same-Frequency Inhibition and Excitation (SFIE) model [13] to study possible neural mechanisms underlying the physiological results. This model is the cascade of a physiologically based AN model, a phenomenological ventral cochlear nucleus (VCN) cell model, and an IC cell model with the same structure as the VCN model (Figure 4a). VCN and IC models receive both excitatory post-synaptic potential (EPSP) and delayed inhibitory PSP (IPSP) in the form of an alpha function. Inhibition at the IC stage is both stronger and slower than at the VCN stage. Key model parameters and the range of values used in the simulation are shown in Table I.

## 3. Results

### 3.1. Non-tonotopic Rate Tuning to ERR

Figure 2a shows the firing rate of a neuron as a function of *F*_0_ for an HCT in COS phase. The rate profile shows multiple peaks occurring when the *F*_0_ is a small integer submultiple of the BF (BF, BF/2, BF/3, …), reflecting cochlear tuning to resolved harmonics. This rate-place code to resolved harmonics is inherited from the auditory nerve [1] and is not the focus of the present paper. In contrast, the rate-*F*_0_ profile for the neuron in Figure 2b showed a single peak at 224 Hz for COS HCT. Though this frequency was near BF/2, there were no peaks at other submultiples of BF. This neuron gave a weak, nearly flat response to RAND HCT. Since RAND and COS stimuli have identical power spectra, the difference in response profile suggests that the neuron’s tuning to COS HCT is unrelated to cochlear frequency selectivity but rather depends on temporal envelope fluctuations. With ALT HCT, the neuron exhibited bandpass tuning similar to COS, but shifted one octave towards lower *F*_0_s. Since the ERR of ALT is twice the *F*_0_, this neuron seems to be tuned to ERR, not *F*_0_.

Among 145 neurons tested with HCT, 32% showed bandpass tuning to ERR as in Figure 2b. We defined the “best ERR” as the *F*_0_ where a bandpass neuron reached its maximum firing rate in response to COS HCT. A “best ERR” was not defined for neurons with other tuning shapes to ERR (5% were band-reject, 6% lowpass, 14% highpass, 4% flat, 39% other).

**Figure 3.**
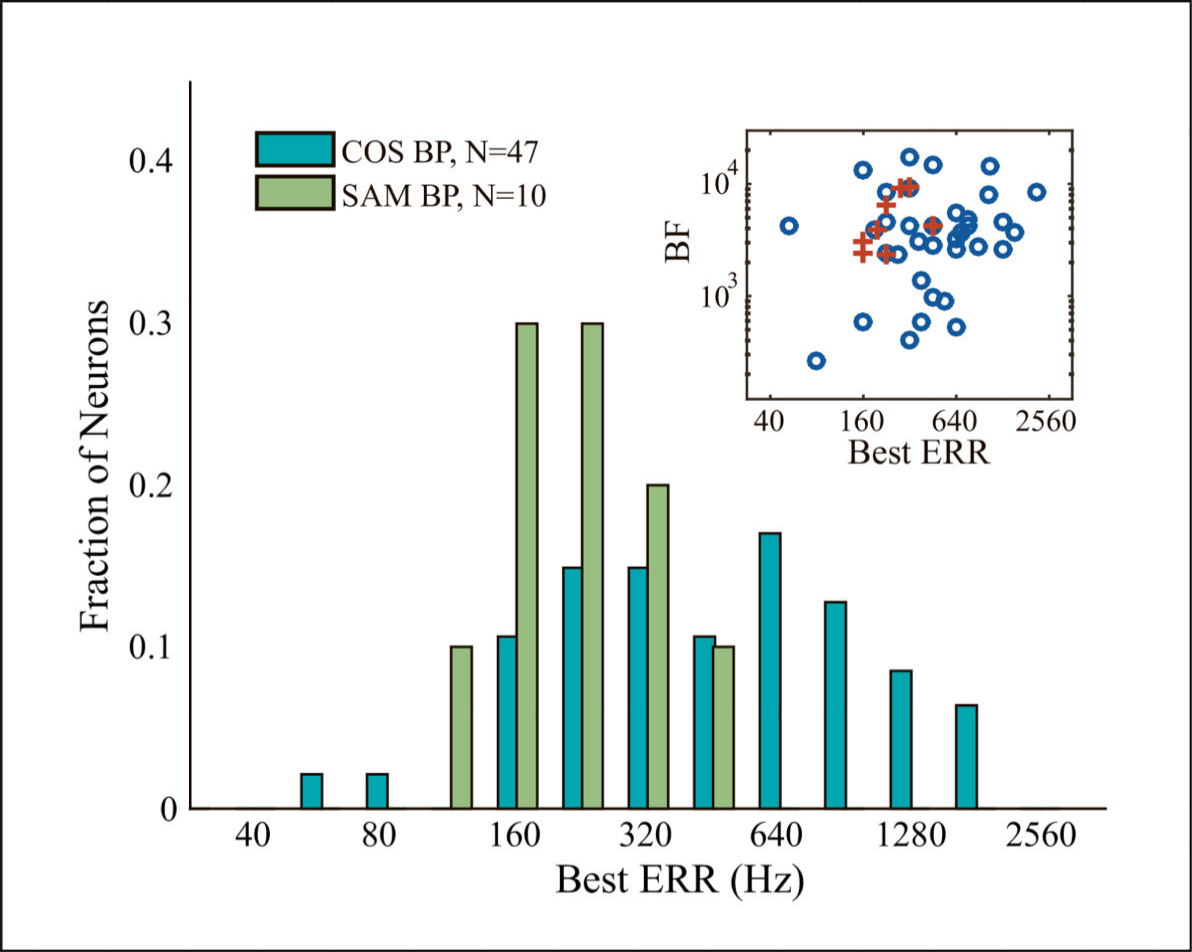
Distribution of best ERR for COS HCT and SAMN. Inset: individual neuronSs best ERR for COS (N = 35, circle) and SAMN (N = 8, cross) showed no correlation with BF. The number of neurons included is smaller than in the histogram because BF could not be identified in some neurons with complex response pattern to pure tones.

Across the neuronal sample, best ERR for COS HCT spanned a wide frequency range, mainly 160–1500 Hz (Figure 3). The inset of Figure 3 shows a scatter plot of the best ERR vs. the pure tone BF for neurons tuned to ERR. There is no correlation between the two frequencies, justifying the term “non-tonotopic tuning” to ERR.

In 66 neurons, we compared rate tuning to ERR for HCT vs. SAMN. As with HCT, we defined the best ERR for SAMN as the *F*_*m*_ yielding the maximum firing rate. The neuron in Figure 2b showed bandpass tuning to the ERR of both COS and SAMN, but the best ERR was lower for SAMN. Among the 66 neurons tested with SAMN, only 10 showed bandpass tuning. While the best ERRs for SAMN were uncorrelated with BF (Figure 3 inset), they were restricted to a lower frequency range (112–448 Hz, Figure 3) than best ERRs for COS. For the 8 neurons showing bandpass tuning to both COS HCT and SAMN, the correlation between best ERR for the two stimuli was not statistically significant (r=0.51, p=0.2).

### 3.2. Model Response to HCT and SAM Stimuli

**Figure 4.**
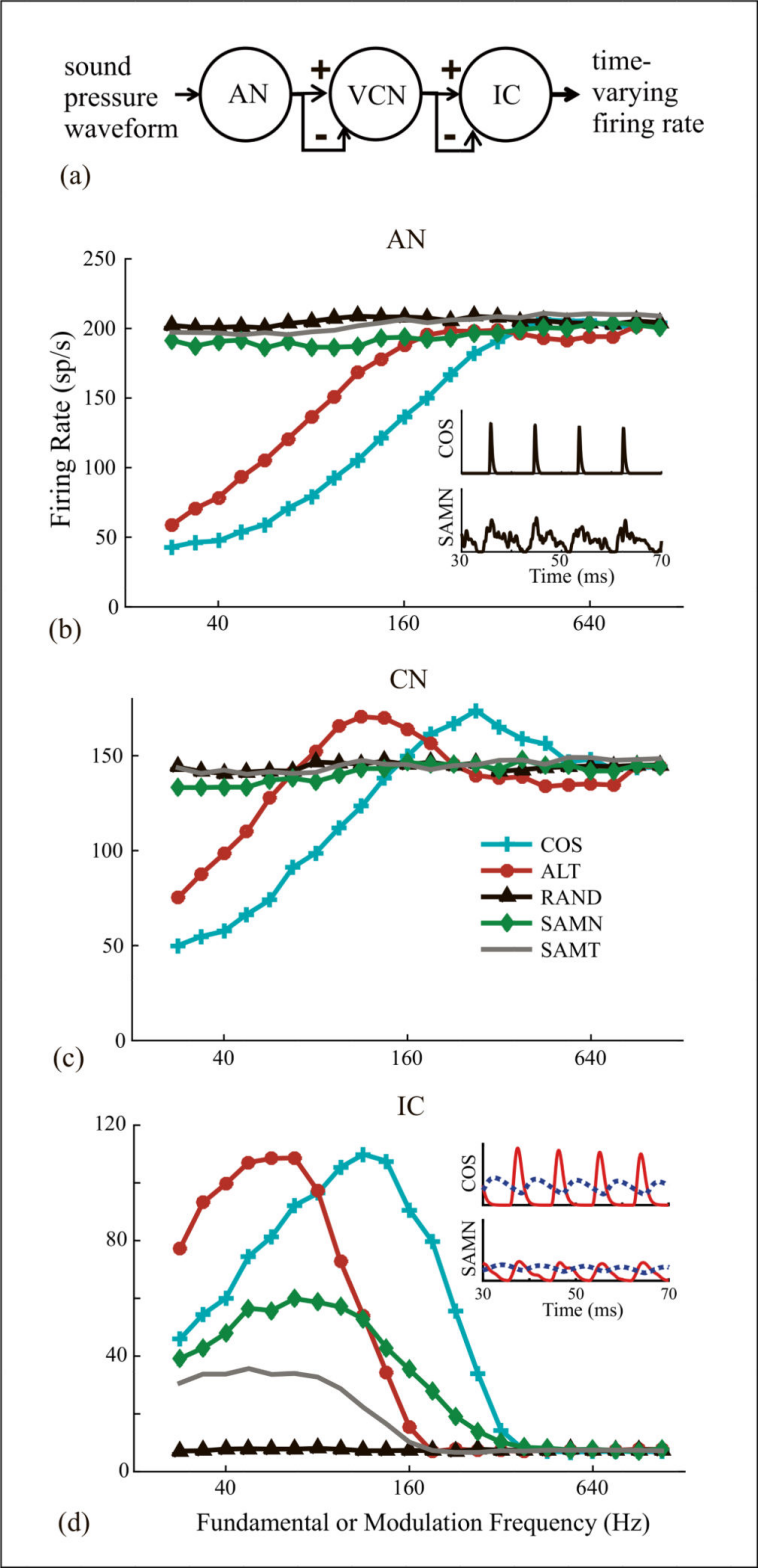
Simulated rate-frequency profiles of SFIE model in response to HCT and SAM stimuli. (a) Model diagram. (b) AN model output. Inset: steady state firing pattern at ERR = 113 Hz for COS HCT and SAMN (SAMN y axis zoomed in 4x). (c) CN model output. (d) IC model output. Inset: steady state EPSP (continuous) and IPSP (dashed) at ERR = 113 Hz. Parameters: BF = 12.8kHz, CN inh_str = 0.6, t_inh_ = 2ms, te_x_ = 0.5 ms, IC inh_str = 1.5, t_inh_ = 3 ms, t_ex_ = 0.5 ms.

To explore possible mechanisms giving rise to the non- tonotopic tuning to ERR, we stimulated the SFIE model with HCT, SAMN and SAM tones (carrier frequency at the model’s BF). Rate-frequency profiles are shown in Figure 4b-d for each model stage. At the AN stage, the model fired approximately 1 spike per ERR cycle for COS and ALT HCT up to 200 Hz, then the rate plateaued at higher frequencies. The rising portion of these profiles was maintained at subsequent stages and contributed to bandpass ERR tuning for COS and ALT at the IC stage. In contrast, AN firing rates for RAND and SAM were nearly constant over the entire frequency range. VCN responses were similar to AN except for a modest overshoot before the plateau with ALT and COS. Model IC responses resembled the neural data in Figure 2b: bandpass tuning to ERR for COS and ALT, and flat, weak response to RAND. Importantly, the model firing rate peaked one octave below for ALT compared to COS, consistent with the neural data. The model showed bandpass tuning for SAMN with a lower best ERR than for COS, consistent with the trends in the neural data.

We simulated the model with various parameter combinations (Table I). Rate tuning to ERR was observed in some model configurations, with best ERR for COS ranging from 48 to 190 Hz (not shown), which is lower than the range in the neural data. A critical factor for ERR tuning was the balance between inhibition and excitation at the IC stage. When inhibition was weaker than excitation, the IC output resembled the VCN response in Figure 4c, with minimal tuning. A decrease in firing rate at high frequencies, which is necessary for bandpass tuning, only occurred when the inhibition was stronger than excitation [13].

To understand the difference in tuning between SAMN and COS HCT observed in both the data and the model, the insets in Figure 4b and 4d show the temporal firing patterns of the AN stage and PSPs of the IC stage at 113 Hz, the model’s best ERR for COS. The temporal firing patterns of the AN stage reflect the different stimulus envelope shapes-transient, impulse-like for COS, and more graded for SAMN. As explained by Nelson and Carney [13], the PSPs of the VCN and IC stages turn from phasic to tonic with increasing input frequency. With COS stimuli at 113 Hz, the transient EPSPs at the input to the IC stage occur out of phase with the nearly sinusoidal IPSP, allowing strong firing. In contrast, the EPSPs for SAMN have lower amplitude, and the IPSP is sustained, resulting in weak firing. Thus, the dynamics of excitation and inhibition in the model interact with the different envelope shapes of COS and SAMN to yield a lower best ERR for SAMN.

## 4. Discussion

### 4.1. Implication for Pitch Processing

We identified a non-tonotopic rate code for ERR of HCT in the IC of unanesthetized rabbits. This finding extends the results in [14], where multi-unit clusters in the IC of anesthetized guinea pigs showed bandpass or band-reject rate tuning to ERR of HCT in sine and ALT phase. Best ERRs in their study ranged from 50 to 400 Hz, with a mode at 141 Hz. This is clearly lower than the 160–1500 Hz range we observed, in part because they only tested *F*_0_ up to 400 Hz, and perhaps also because they used an anesthetized preparation.

Tuning to ERR could be reproduced by the SFIE model, thereby suggesting a mechanism for how the peripheral temporal code for ERR is transformed into a rate code in the midbrain. However, ERR and pitch are not equivalent. For example, the pitch of an ALT HCT containing resolved harmonics is matched to its *F*_0_, not ERR [9]. Nevertheless, the non-tonotopic rate code for ERR could play a role in extracting the pitch of unresolved harmonics. This information could be integrated with the more reliable pitch information provided by resolved harmonics at a central “pitch center”. The non-tonotopic rate code may be especially important in listeners with sensorineural hearing loss, where reduced cochlear frequency selectivity results in a degraded representation of resolved harmonics [15].

### 4.2. ERR Tuning Depends on Envelope Shape

The differences in ERR tuning between HCT and SAMN suggests that the tuning is dependent on envelope shape, consistent with previous studies in anesthetized cat [16], chinchilla [17] and gerbil [18, 19] that also found IC neurons respond differently to AM stimuli with various envelope shapes. The non-tonotopic rate code to ERR in IC, therefore, is confounded by the sensitivity to envelope shape.

### 4.3. Limitations of SFIE Model

The SFIE model was able to simulate non-tonotopic rate tuning over a range of best ERRs for COS HCT. However, the range of best ERR that could be generated in the model was restricted to lower frequencies (48–190 Hz) compared to the neural data (160–1500 Hz). Because the ERR tuning is dependent on the transient, synchronized response of AN and CN stages, adding multiple input pathways to IC might enhance the strong fluctuations of PSPs at high frequencies, thereby extending the range of best ERR. As expected, the model only simulated bandpass tuning; band-reject and other complex response patterns were not reproduced. More recent elaborations of the SFIE model with more complex structures can account for other tuning types with SAM stimuli [20], although they have not yet been systematically tested with HCT.

## Figures and Tables

**Table I.** Key VCN/IC model parameters.

	VCN	IC
Inhibition delay re. excitation (ms)	1–3	1–8
EPSP time constant *τ*_ex_ (ms)	0.3–0.5	0.5–2
IPSP time constant *τ*_inh_ (ms)	0.5–2	0.5–10
inhibition strength re. excitation inh_str	0–1.8	0–1.8
